# Dynamic changes in platelet counts and psychological state in ITP patients after COVID-19 infection

**DOI:** 10.3389/fmed.2025.1485418

**Published:** 2025-03-14

**Authors:** Xiaoli Chen, Dan Wang, Jie Tang, Li Wang, Yao Quan, Jia Liu, Zhongmin Zou, Hengrui Sun, Yimei Feng, Xi Zhang

**Affiliations:** ^1^Medical Center of Hematology, The Xinqiao Hospital of Army Medical University, Chongqing, China; ^2^Department of Hematology, The General Hospital of Western Theater Command, PLA, Chengdu, China; ^3^Department of Chemical Defense Medicine, School of Military Preventive Medicine, Army Medical University, Chongqing, China

**Keywords:** ITP, COVID-19, platelet counts, psychological state, PLR

## Abstract

**Introduction:**

At the end of 2022, the COVID-19 wreaked havoc in China as well as around the world. Here, we focused on the dynamic changes in platelet kinetics and psychological state in ITP patients before and after infection with COVID-19.

**Methods:**

A questionnaire survey was designed to retrospectively investigate the COVID-19-related symptoms, changes in platelet count, and psychological changes in ITP patients infected with Omicron variant during November 2022 to January 2023, with a healthy population survey conducted as a control.

**Results:**

A total of 90 ITP patients and 69 healthy individuals were included in the study. The results showed that only in terms of low-grade fever, the proportion of ITP patients was significantly higher than that of healthy individuals, 31% vs. 17% (*p* = 0.04). Interestingly, it was found that there was a transient elevation in platelet counts (PC) of ITP patients after COVID-19 infection, which then gradually decreased to the previous level after recovering from virus, including three subgroups comparation: PC >100 × 10^9^/L vs. PC <100 × 10^9^/L; ITP treatment vs. non-ITP treatment; and vaccination vs. non-vaccination. Additionally, the platelet-to-lymphocyte ratio (PLR) showed the same trend. The fear and concerns related to COVID-19 infection were also compared between the two population. For ITP patients, they are more concerned that COVID-19 will worsen the condition of ITP and delay its recovery. It should be pointed out that the limitations of this study include the small sample size in the retrospective survey and the possibility of selection bias in ITP patients.

**Conclusion:**

Some ITP patients showed transiently elevated platelet counts after COVID-19 infection, and the specific mechanism requires further exploration. Additionally, ITP patients experienced heightened anxiety and tension after COVID-19 infection, needing for more mental health support.

## 1 Introduction

The outbreak of the 2019 coronavirus disease (COVID-19) quickly became a major global infectious disease (1), demonstrating it was the most intractable variant, although COVID-19 vaccines greatly reduce the severity and complications of SARS-CoV-2 infection (2). The Omicron variant emerged in human populations in November 2021, and its transmissibility and immune evasion capability have significantly increased compared to other variants. It rapidly replaced the Delta variant in early 2022 and became the globally dominant epidemic strain (3, 4).

With the updates and changes in the COVID-19 prevention & control policies implemented by the Chinese government by the end of 2022, a fraction of people including immune thrombocytopenia (ITP) patients were infected with the Omicron variant of the virus. The clinical manifestations of COVID-19 vary widely, and hematological changes are common, including decreased lymphocyte and platelet counts (PCs) (5, 6). Therefore, for patients with blood disorders, a COVID-19 infection can worsen the severity of their underlying condition and even pose a threat to their life.

As we know, ITP can be induced or associated with COVID-19 infection (7–10). Few studies have examined the dynamic changes in platelet levels in ITP patients after COVID-19 infection, and there are currently no reports on the psychological changes in these patients. A recent report suggests that platelets may temporarily increase in ITP patients following such an infection (11). In our ITP patients, we also observed similar phenomena. Hence, our study focused on the population of patients with immune thrombocytopenia, and we were curious whether ITP patients, after contracting the Omicron variant, experience any ITP-related abnormal symptoms compared to healthy individuals. Specific to our interest, were there any changes in platelet counts and psychological status among ITP patients? Due to the retrospective nature of the study, there may be selection bias among ITP patients and healthy individuals.

## 2 Data and methods

### 2.1 Patient data

By conducting a questionnaire survey, this study retrospectively investigated and analyzed the COVID-19-related symptoms, platelet changes, and fear and worry scores of psychological evaluations among the ITP population who were infected with COVID-19 during the period of November 2022 to January 2023. A survey was also conducted on a healthy population as a control group. This study was approved by the Ethics Committee of Xinqiao Hospital, Army Medical University.

### 2.2 Diagnostic criteria

The diagnostic criteria for ITP were as follows: (1) blood routine examination conducted at least twice with a platelet count (PC) of less than 100 × 10^9^/L and without abnormal blood cell morphology. (2) Presence or absence of clinical manifestations such as body bruises, skin bleeding, mucosal bleeding, or internal bleeding. (3) Typically, no splenomegaly. (4) Bone marrow examination displaying increased or normal number of megakaryocytes with maturation disorders. (5) Exclusion of other secondary causes of thrombocytopenia, such as inherited or acquired blood disorders, leukemias, myelodysplastic syndromes with isolated thrombocytopenia, or thrombocytopenia associated with other immune disorders or infections (12).

According to Diagnosis and Treatment Plan of SARS-CoV-2 Infection (Trial Version 10 issued by National Health Commission of China), diagnosis was made through a comprehensive analysis of epidemiological history, clinical manifestations, and laboratory tests. The primary criterion for diagnosis was a positive result in nucleic acid testing for the novel coronavirus infection. The diagnostic criteria were as follows: (1) clinical manifestations of COVID-19 infection. (2) Presence of one or more of the following etiological and serological examination results: (1) positive for COVID-19 nucleic acid test; (2) positive for COVID-19 antigen test; (3) positive for COVID-19 virus isolation and culture; (4) acute-phase specific IgG antibody level against COVID-19 in the recovery period is 4 times or more elevated (13).

### 2.3 Treatment methods

In this study, ITP patients were further divided into three groups: the patients who did not meet the treatment standards and had not yet started medication, and patients were currently undergoing ITP treatment; and the patients had completed medication and were under observation. The ITP treatment was conducted based on the Chinese ITP guidelines and our center’s experience, including traditional Chinese medicine, glucocorticoids (dexamethasone, prednisone), TPO receptor agonists (TPO-RAs), cyclosporine, rapamycin, etc. (12, 14).

### 2.4 Design and implementation of emotional scale

To evaluate psychological status of ITP patients, a questionnaire was designed with reference to the COVID-19 related mental scale (15, 16), including the following items: (1) General conditions: age, sex, education, marital status, course of illness, comorbidities, etc.; (2) COVID-19 infection related Fear Scale consisted of seven subjects, scored on a 5-point scale from 1 (strongly agree) to 5 (strongly disagree). The average score indicated the level of fear regarding COVID-19 infection, with higher scores indicating a greater level of fear. (3) COVID-19 infection related Anxiety Scale had 10 items, scored on a 5-point scale from 1 (not strong) to 5 (very strong). Similarly, the average score indicated the level of anxiety and worry, with higher scores meaning higher levels. Medical staff evaluated the enrolled patients and guide them to complete the questionnaire survey on depression, anxiety, depression, and sleep status. The original scale had a Cronbach’s *α* coefficient of 0.82 (15), and the Cronbach’s *α* coefficient of the scale in this study was 0.855, demonstrating good reliability.

### 2.5 Statistical analysis

Statistical analysis was performed using SPSS 22.0 and GraphPad Prism 6. The enumeration data was expressed as percentage (%), and the measurement data was expressed as ( $\bar{x\;}\pm s$ ). ANOVA (normal distribution) or nonparametric test (non-normal distribution) for continuous variables and the chi-square test was used to analyze the clinical features of patients. *p* < 0.05 was statistically significant. Multivariate analysis was performed using R 4.2.1 (http://www.Rproject.org; The R Foundation, Vienna, Austria) and the Free Statistics software (version 1.9.2; Beijing FreeClinical Medical Technology Co., Ltd., Beijing, China). Missing covariate data are replaced with statistical estimates of missing values, missing values for continuous variables are taken as means, medians, or patterns, and missing values for categorical variables are treated as a separate group.

We primarily utilized descriptive statistics to illustrate the variations in platelet counts among ITP patients before and after COVID-19 infection. This method allows for straightforward data visualization without complex statistical inference. Anyway, to control the potential confounders, we categorized ITP patients based on platelet count levels before infection (PC <100 × 10^9^/L and PC ≥100 × 10^9^/L), vaccination status (vaccinated vs. unvaccinated), and treatment for ITP (treated vs. untreated). This stratification method aids in assessing changes in platelet counts across different subgroups, thereby reducing potential confounding effects, and these categories are also of clinical significance. In the subsequent analysis, we incorporated a multifactorial analysis to better control for various factors, including age, sex, BMI, and ITP treatment, etc.

## 3 Results

### 3.1 Basic characters of ITP patients

A total of 90 ITP patients were included, all of whom were infected with the coronavirus and have recovered. The average age was 40 (12–67) years old. Males accounted for 83% (75/90) and females for 17% (15/90). Patients over 60 years old took a proportion of 11% (10/90) of the total, while patients under 60 years old of 89% (80/90). There were 62 patients currently undergoing ITP-related treatment, 22 people who had finished medication and were under observation, and six people who had never received any treatment. The median course of ITP duration was 65 months (6–278 months), with a median of 2 (1–4) prior treatment lines received, and a median of 1 (1–3) occurrences of COVID-19 infection. The median time for the coronavirus to turn negative was 7 (3–11) days. About vaccination, 50 patients had received COVID-19 vaccines and 40 patients not subjected (Table 1).

**Table 1:** Basic characters of ITP patients.

Parameter	Value
Age (range)	
Median age	40 (12–67)
≥60 years	11.1% (10/90)
<60 years	88.9% (80/90)
Gender	
Male	16.7% (15/90)
Female	83.3% (75/90)
BMI	
Mean BMI	32.1 ± 0.4
Normal (18–24)	33.3% (30/90)
Abnormal (<18 and >24)	66.7% (60/90)
Median time from diagnosis of ITP (months)	65 (6–278)
Median treatment lines of ITP	2 (1–4)
ITP treatment plan	
The untreated	6.7% (6/90)
Drug withdrawal	24.4% (22/90)
The treated	68.9% (62/90)
COVID-19 vaccination	
Vaccinated	55.6% (50/90)
Not vaccinated	44.4% (40/90)
The median time for COVID-19 nucleic acid to negative (days)	7.2 (3–11)

### 3.2 Comparison of COVID-19 related symptoms between ITP patients and healthy people

**Figure 1:**
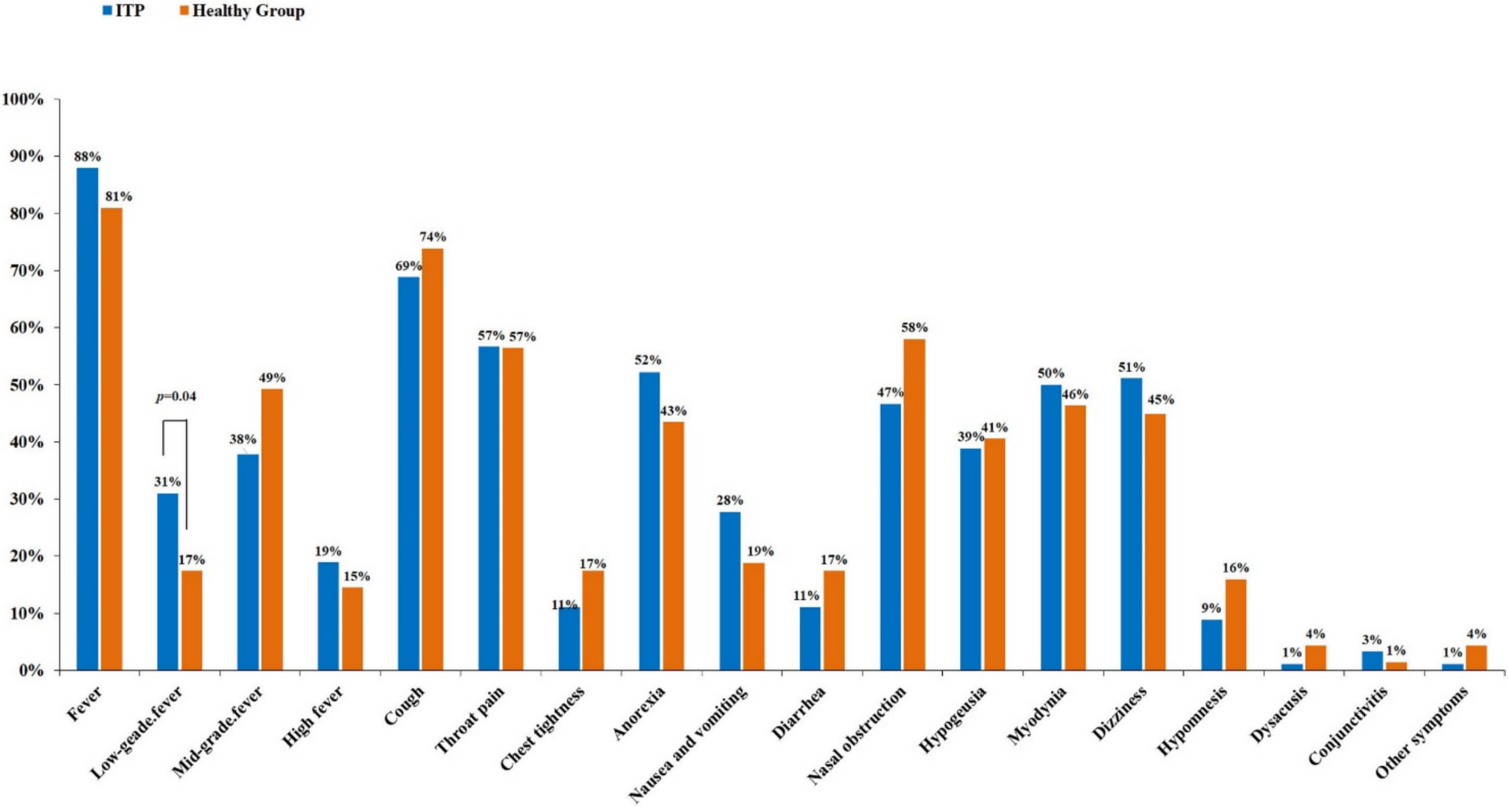
Comparison of symptoms related to COVID-19 infection between ITP and healthy people. In comparing 19 common symptoms, individuals with ITP have a higher incidence of low-grade fever than healthy individuals, with a statistically significant difference (*p* = 0.04), while other symptoms do not show significant differences.

A control group of 69 healthy individuals who were infected with the coronavirus and recovered were included. We compared the differences in related symptoms after COVID-19 infection between the two groups. Common clinical symptoms were recorded, including fever (low-grade, moderate-grade, high-grade), cough, throat pain, chest tightness, difficult breathing, poor appetite, nausea and vomiting, diarrhea, etc. The results have shown that the proportion of fever was over 80% in both the ITP patients and the healthy group, making it the highest proportion among all the symptoms. In terms of low-grade fever, the proportion among ITP patients was significantly higher than that in the healthy group, with 31% vs. 17% (*p* = 0.04). There were no statistical differences between the two groups regarding the remaining symptoms (Figure 1) (Supplementary file 1).

### 3.3 Changes in platelet count after COVID-19 infection

**Figure 2:**
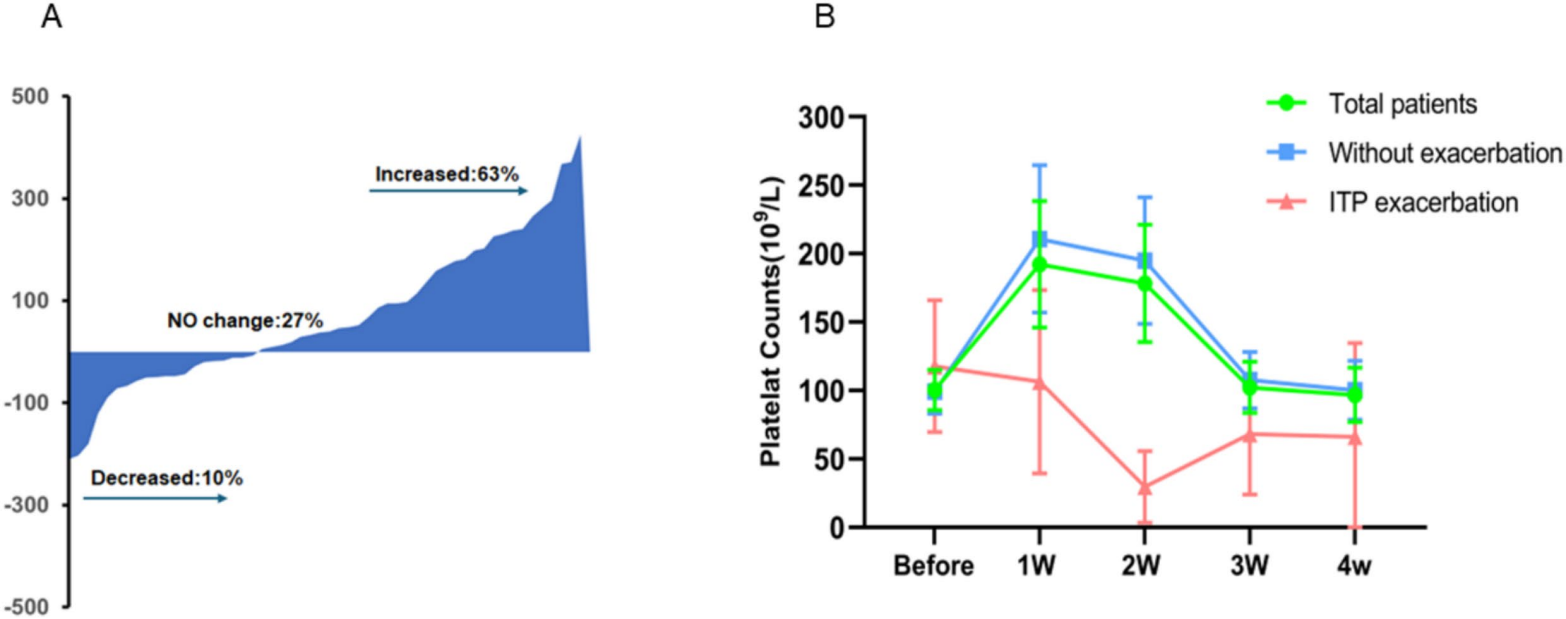
Dynamic changes in platelet counts from COVID-19 onset to follow-up. In ITP patients, platelet counts can either increase (63%), decrease (10%), or remain unchanged (27%) after COVID-19 infection, with increases being the most common **(A)**. Overall, platelet counts in these patients tend to rise temporarily following the virus infection, peaking in the first week, and generally return to baseline levels approximately 3 weeks post-recovery **(B)**.

Both baseline and 2-week platelet counts were available for 90 ITP patients. If the change was defined as a >20% fluctuation, 9 (10%) patients showed a decrease, 24 (27%) patients showed no change, and 57 (63%) patients showed an increase in the platelet count at 2 weeks after COVID-19 infection (Figure 2A).

In general, platelet counts of 90 ITP patients were measured before and after being infected with COVID-19. According to statistical results, the mean platelet counts before infection was 100 × 10^9^/L. The mean PC 1 week after recovery from infection was 192 × 10^9^/L, 2 weeks after recovery was 178 × 10^9^/L, 3 weeks after recovery was 102 × 10^9^/L, and 4 weeks after recovery was 97 × 10^9^/L. Meaningfully, the increased PC 1 week after recovery from the coronavirus infection was statistically significance compared to the PC level before COVID-19 infection (*p* = 0.000) (Figure 2B). The dynamic changed of PC after COVID-19 infection could be depicted as a transient increase in 1–2 weeks before a gradual decline over the following 2 weeks to the pre-infection level.

### 3.4 Dynamic PC changes based on initial PC level

**Figure 3:**
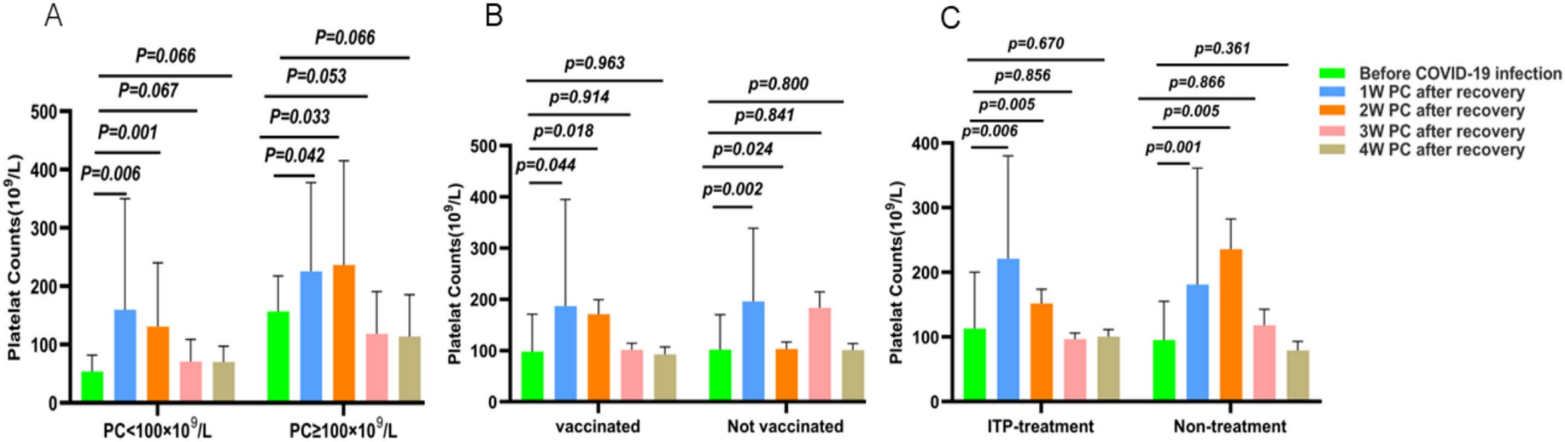
Trend of changes in platelet counts in subgroup analysis. The three subgroups (**A**: CR group vs. no CR group; **B**: vaccinated group vs. not-vaccinated group; **C**: ITP treatment group vs. not treated group) showed a similar trend after infection with COVID-19. Platelets initially increased and then decreased. The platelet counts generally peaked 1 week after recovery from COVID-19, whereas it peaked at 2 weeks in the untreated group and the CR group.

Further, we categorized patients into two groups based on their platelet counts before contracting the virus (Group A: PC <100 × 10^9^/L and Group B: PC ≥100 × 10^9^/L). For Group A, the average platelet count prior to COVID-19 infection was 54 × 10^9^/L. In the initial week post-infection, the average PC rose to 160 × 10^9^/L. Subsequently, it dropped to 131 × 10^9^/L in the second week, further declining to 71 × 10^9^/L by the third week, and stabilizing at 70 × 10^9^/L in the fourth week. As for Group B, the mean platelet counts pre-COVID-19 infection stood at 155 × 10^9^/L. Within the following 4 weeks of infection, the mean PC surged to 226 × 10^9^/L, peaked at 236 × 10^9^/L, then decreased to 119 × 10^9^/L, ultimately settling at 114 × 10^9^/L. Notably, regardless of whether the PC was above or below 100 × 10^9^/L, the overall pattern remained consistent, with a notable disparity in platelet counts before and 1 week after COVID-19 infection (*p* < 0.05). However, in Group A, platelet counts started declining in the initial week, while in Group B, this decrease commenced after 2 weeks. Ultimately, after a 3 to 4-week recovery period following COVID-19 infection, platelet counts returned to baseline levels (Figure 3A).

### 3.5 Dynamic PC changes based on vaccination status

Next, the patients were analyzed based on whether they were vaccinated against the COVID-19 (Vaccinated and Not vaccinated). The baseline (before infection) mean PC of vaccinated patients was 98 × 10^9^/L, after the virus antigen test turned negative, the PC at weeks 1, 2, 3, and 4 were 187, 171, 101, and 93 × 10^9^/L, respectively. Similarly, the average PC of patient without vaccination before COVID-19 infection was 102 × 10^9^/L. In weeks 1, 2, 3, and 4 after the patients recovered, the PC were 196, 183, 103, and 101 × 10^9^/L, respectively. Whether being vaccinated or not, the trend of platelet changes indicated an initial increase followed by a decline. Upon analysis, it was observed that there was a notable and statistically significant difference in platelet counts between the pre-infection period and the initial week of recovery for both groups (*p* = 0.044, and *p* = 0.002) (Figure 3B).

### 3.6 Dynamic PC changes based on ITP treatment

In addition, we divided the ITP patients into two groups based on whether they were receiving ITP treatment (ITP treatment and non-ITP treatment). The average platelet counts before COVID-19 infection in non-ITP treated patients was 95 × 10^9^/L. In the first week after recovering from the viral infection, the average platelet count increased to 181 × 10^9^/L. In the second week, it slightly increased to 236 × 10^9^/L. In the third week, it further decreased to 118 × 10^9^/L, and in the fourth week, it reached 79 × 10^9^/L. For patients underlying ITP treatment, the platelet count before COVID-19 infection was 113 × 10^9^/L. In the first week after recovery, the mean platelet count was 221 × 10^9^/L, in the second week it was 152 × 10^9^/L, in the third week it was 97 × 10^9^/L, and in the fourth week it was 101 × 10^9^/L. The analysis results revealed that trend of PC changes was as the same with the subgroup outcomes. Interestingly, the platelet counts of the non-ITP treatment group began to decrease from the third week after infection recovery, whereas the ITP treatment group’s platelet count began to decrease from the second week after COVID-19 recovery (Figure 3C).

In the subsequent analysis, we incorporated a multifactorial analysis to better control for various factors, including age, sex, BMI, and ITP treatment. The results showed that there was a significant negative correlation between the platelet count before COVID-19 infection and the clearance time of SARS CoV-2 virus (*p* = 0.021). However, no correlation was found between platelet variations and factors such as age, sex, and BMI. These findings enhance the reliability of our results and contribute to understanding the complexities surrounding platelet count changes in ITP patients before and after COVID-19 infection (Supplementary file 2).

In summary, we categorized ITP patients based on whether they received ITP-related treatment, their response status (CR or non-CR), and vaccination status. The consistent outcome was that the average platelet count increased during the first week after COVID-19 infection, then gradually decreased, returning to baseline levels by the third week. However, potential mechanisms have not been further investigated, including the lack of measurement of levels of IL-6, TPO, or lymphocyte subgroups such as T, B, and NK cells.

### 3.7 Psychological changes of ITP patients after COVID-19 infection

In addition to analyzing symptoms and platelet changes after COVID-19 infection, we separately assessed the level of psychological fear of COVID-19 infection in healthy individuals and patients with ITP.

According to the questionnaire results, it was found that both groups scored above 3 in terms of fear of contracting COVID-19 and the possibility of the virus causing lingering effects or even death, indicating a high level of fear among the participants. However, the overall mean scores for both groups were below 3 in the other five items survey, suggesting a relatively low level of fear towards the current COVID-19 pandemic. There was no statistically significant difference between ITP patients and healthy individuals (Table 2).

**Table 2:** Fear scale for two groups of people.

Survey item	ITP patients	Healthy people	*p*-value
	Average score	Average score	
I am very afraid of the COVID-19 infection	3.1 ± 0.128	3 ± 0.110	0.304
I am afraid of that COVID-19 with leave sequelae or even cause death	3.2 ± 0.142	3.2 ± 0.133	0.927
When I think of the COVID-19, my hands get wet	2.0 ± 0.109	2.2 ± 0.106	0.195
Whenever I think about COVID-19, I feel uncomfortable	2.9 ± 0.129	2.8 ± 0.129	0.781
I feel nervous or anxious when getting news and stories about COVID-19 on social media	2.9 ± 0.124	2.8 ± 0.115	0.752
I cannot sleep well for fear of COVID-19	2.1 ± 0.108	2.2 ± 0.114	0.543
When I think of the COVID-19, my heart beats faster or has palpitations	2.3 ± 0.112	2.6 ± 0.117	0.135
Total average score	2.6 ± 0.124	2.3 ± 0.113	0.301

Based on the published Fear Scale, we designed a 10-item worry scale for individuals who have been infected with the coronavirus, and collected feedback from ITP patients and healthy individuals. Finally, we evaluated the scores for each item for each patient. It was found that both groups had clear concerns about transmitting the virus to older adults or children, with healthy individuals expressing stronger concerns. For ITP patients, there were strong concerns about “Worsening complications such as organ bleeding,” “Increasing caregiving pressure to the family” and “Affecting platelet recovery” after contracting the virus. And for the three aspects, there was a significant statistical difference in the mean scores for the 10 worries between the two groups (*p* < 0.001) (Table 3).

**Table 3:** Worry scale for two groups of people.

If you are infected with the COVID-19 (or have been infected), the concerns you may have been as follows	ITP patients	Healthy people	*p*-value
	Average score	Average score	
Left with sequelae	2.7 ± 0.156	3 ± 0.167	0.156
Transmitted to parents (elderly)	2.9 ± 0.154	3.5 ± 0.182	0.007
Transmitted to children (young)	2.8 ± 0.157	3.5 ± 0.191	0.002
Being socially discriminated	1.7 ± 0.102	1.9 ± 0.132	0.174
Worsening complication such as organ bleeding	3.1 ± 0.155	1 ± 0.000	*p* < 0.001
Increasing caregiving pressure to the family	3 ± 0.156	1 ± 0.000	*p* < 0.001
Impacting on work or lowering academic performance	2.9 ± 0.146	3 ± 0.000	0.923
Affecting platelet recovery	3.5 ± 0.144	1 ± 0.000	*p* < 0.001
Worried about being infected again	3.5 ± 0.145	3.2 ± 0.181	0.137
Other concerns	2.4 ± 0.154	2.3 ± 0.154	0.814
Total average score	2.6 ± 0.157	2.3 ± 0.153	0.046

Our study focuses on the psychological changes of ITP patients regarding COVID-19 and their concerns about ITP disease, which is a distinctive feature of this research. Table 2 was designed to compare the awareness of COVID-19 among ITP patients and healthy individuals, revealing no significant difference in overall scores (*p* = 0.301). This suggests that as understanding of COVID-19 deepens, fear of the disease decreases. However, ITP patients express significant concerns about the impact of COVID-19 on their condition if infected, such as exacerbated organ bleeding, delayed platelet recovery, and increased caregiving burden (Table 3). Statistically, the two groups show a significant difference in concerns about the exacerbation of ITP symptoms (*p* = 0.046). Additionally, while our questionnaire was primarily aimed at understanding the psychological effects of the Omicron outbreak, it did not extensively address the long-term impacts of COVID-19 on ITP patients. We believe that the psychological effects of persistent COVID-19 related symptoms or repeated COVID-19 infections in ITP patients merit further investigation.

### 3.8 Evaluation of the hematological inflammatory parameters before and after viral infection

Hematological inflammatory parameters of ITP patients, including monocyte-to-lymphocyte ratio (MLR), neutrophil-to-lymphocyte ratio (NLR) and platelet-to-lymphocyte ratio (PLR), were evaluated before and after viral infection. Before the COVID-19 infection, the median PLR was 54.0 (30.2, 77.3), but after viral infection, the PLR increased to 83.6 (41.1, 127.5) (*p* = 0.001). By the third week, it was down to 59.4 (32.6, 96.4) (*p* = 0.980). However, the MLR and NLR showed no significant increase or decrease before and after the viral infection, remaining relatively stable (Table 4).

**Table 4:** Hematological inflammatory parameters before and after viral infection.

Index	Median1	Median2	*p*	Median3	*p*	Median4	*p*
MLR	0.2 (0.2, 0.3)	0.2 (0.2, 0.3)	0.368	0.2 (0.2, 0.3)	0.980	0.2 (0.2, 0.3)	0.899
NLR	2.1 (1.5, 3.1)	2.3 (1.8, 3.2)	0.636	2.2 (1.7, 2.9)	0.717	2.1 (1.6, 3.0)	0.966
PLR	54.0 (30.2, 77.3)	83.6 (41.1, 127.5)	0.001^*^	50.5 (21.7, 84.2)	0.039^**^	59.4 (32.6, 96.4)	0.980

MLR, monocyte-to-lymphocyte ratio; NLR, neutrophil-to-lymphocyte ratio; PLR, platelet-to-lymphocyte ratio; Median1, before viral infection; Median2, 1 week after recovery; Median3, 2 weeks after recovery; Median4, 3 weeks after recovery. Paired *t*-test is used for statistical analysis. ^*^p = 0.001 indicates a statistically significant difference in PLR between the ITP population before COVID-19 infection and one week after recovery from the viral infection. ^**^p = 0.039 indicates a statistically significant difference in PLR between the ITP population before COVID-19 infection and two weeks after recovery from the viral infection.

## 4 Discussion

ITP can be induced or associated with many viruses, including hepatitis C virus, human immunodeficiency virus, cytomegalovirus, EB virus and severe acute respiratory syndrome coronavirus-1 as well. Severe acute respiratory syndrome coronavirus-2 (SARS-CoV-2) infection leads to excessive activation of inflammation, causing thrombocytopenia (17, 18). In the mechanism of virus-induced thrombocytopenia, 36% of cases are due to increased peripheral platelet destruction caused by excessive activation of inflammatory cytokine storm (19, 20). Thrombocytopenia is detected in 5–41.7% of COVID-19 patients (7), however, there are very few reports about platelet counts increasing rather than decreasing after a COVID-19 infection. Additionally, COVID-19 infection also brings greater psychological pressure to ITP patients due to its world-wide coverage. Patients often worry that COVID-19 infection will worsen their underlying ITP condition. As far as we know, this retrospective study is the first review and analysis of the changes in platelet dynamics, psychological and sleep status of ITP patients following COVID-19 infection, as well as the changes in COVID-19-related symptoms compared to healthy individuals.

The main clinical manifestations of COVID-19 infection are low-grade fever, cough, nasal congestion, mild headache, sore throat, muscle pain, and some patients may experience digestive symptoms such as diarrhea. Laboratory examinations may reveal decreased white blood cell count, elevated calcitonin, and abnormalities such as decreased lymphocyte ratio and elevated C-reactive protein. These symptoms and laboratory abnormalities suggest that COVID-19 infection can cause damage to multiple body systems, which is of great clinical significance in the diagnosis and treatment of the disease. In a retrospective study by Guan et al. (21), fever (87.9%) and cough (67.7%) were the most common symptoms, while diarrhea (3.7%) and vomiting (5.0%) were less common. We observed a similar phenomenon in our study, and ITP patients were more likely to have low-grade fever, which may be due to immune dysfunction and decreased resistance caused by the interference of the underlying condition. Further research is needed to determine the specific mechanisms.

The COVID-19 can cause a decrease in platelet count through various mechanisms. The Spanish ITP expert group (8) believes that virus-induced autoimmunity is one of the important mechanisms leading to decreased platelet count in patients; secondly, the ACE2 receptors on the surface of liver cells can be engaged and destroyed by SARS-CoV-2, leading to a decrease in thrombopoietin (TPO) synthesized in liver cells (9); in addition, antibodies and immune complexes can attach to platelet surfaces in patients, leading to increased platelet destruction caused by phagocytosis in the reticuloendothelial system. On the other hand, patients with fever after COVID-19 infection will develop numerous microthrombi in their lungs. The virus can increase platelet consumption by activating the coagulation cascade in lung endothelial injury (22), or make platelet production ineffective by infiltrating the bone marrow niche. Studies have shown that excessive activation of inflammation cytokine storm leads to increased destruction of peripheral platelets (23), resulting in a gradual decrease in platelet count in ITP patients after COVID-19 infection. Thrombocytopenia is common during COVID-19 infection, but there are also studies that have found rare late or delayed-onset thrombocytopenia (occurring 14 days after symptom onset). A retrospective single-center study recruited 271 patients from Wuhan Union Hospital in China, and among them, 11.8% of the patients experienced COVID-19-related delayed-onset thrombocytopenia. The bone marrow aspirate pathology of three patients with delayed-onset thrombocytopenia was provided and described, showing impaired megakaryocyte maturation. It was speculated that immune-mediated platelet destruction may explain the delayed-onset thrombocytopenia in this group of patients (24).

Not all ITP patients experienced a decrease in platelet count after infecting COVID-19. Research indicated that only 12.5% of ITP patients showed exacerbated platelet, while 34.6% experience no significant change, in contrast, 52.9% of ITP patients had elevated platelets (11). Our study also showed similar results, with (63%) over half of the patients experiencing elevated platelet levels. Previous studies have found that in severe COVID-19, platelet count increases significantly due to cytokine storm. However, the exact mechanisms are still unclear (25, 26). In our follow-up observation of 90 ITP patients, we found that platelet count briefly increased after COVID-19 infection but gradually decreased to baseline levels as the patients recovered from COVID-19. A review of relevant literature found that reactive thrombocytosis was also reported in patients during the 2003 SARS epidemic (27) and 2011 H1N1 infection (28). In a case report, a 55-year-old female patient diagnosed with novel coronavirus pneumonia had a normal platelet count at admission, but it gradually increased to over 1,000 × 10^9^/L with clinical deterioration (29). Tafazoli’s et al. (30) study suggested that not all COVID-19 patients have thrombocytopenia, and a minority of patients show thrombocytosis. They believe that platelet pathology is more complex than activation or hyperactivation, especially due to the role of platelets in inflammation.

Some potential mechanisms behind the transient platelet increase we can discuss. In Castleman disease (31), gastrointestinal tumors (32) and myeloproliferative neoplasms (33), a significantly positive correlation was found between elevated platelet counts and serum IL-6. IL-6 elevation widely exists in COVID-19 infected patients (34, 35), that may increase the platelet counts. In addition, IL-6/STAT3 signaling pathway activation mediated thrombopoietin (TPO) production (36), elevated TPO levels are also detected in patients with COVID-19 (37) and TPO is known to promote platelet production. Another hypothesis maybe lymphopenia, both T and B lymphocyte decreases were detected in COVID-19 patients (38), that may also reduce the damage to platelets in ITP patients infected with COVID-19. Another speculation is that when infected with COVID-19, the immune system focuses on attacking the virus, which might reduce damage to platelets, similar to the strategy of the ancient Chinese Art of War by Sun Tzu: Besiege Wei to Rescue Zhao. All the speculations about cytokine-mediated pathways (e.g., IL-6, TPO signaling) or immune system changes (e.g., T cell activity) that may explain transient platelet changes in our report, anyway, the specific mechanisms are currently unknown and require further research, which is the limitation of this study. By the way, the impact of COVID-19 vaccines on platelet dynamics in ITP patients was reported by our group previously (2). Therefore, this article does not provide a detailed report. Overall, ITP patients may experience worsening platelet levels after receiving certain types of COVID-19 vaccines, but most can recover with active treatment (2).

The psychosocial effects of the COVID-19 pandemic constitute a global challenge for health care. Having a chronic illness, such as ITP, appears to increase the probability of suffering anxiety (39). ITP patients faced with fear, sadness, and anxiety due to the possibility of being infected with SARS-CoV-2 reduced the platelet count, with detrimental consequences on their health (40). Our investigation also reached a similar conclusion. Therefore, psychological health interventions are crucial for ITP patients experiencing elevated anxiety levels during viral outbreaks. To preserve their psychological well-being and reduce their fears, interactive methods based on positive communication should be adopted (41).

Unfortunately, no dynamic multiple psychological evaluations were performed in this study, resulting in a lack of comparative data regarding psychological assessments before and after the increase in platelet levels. Therefore, we cannot determine whether the temporary increase in platelet count is associated with increased anxiety or fear scores. However, according to our survey report and clinical experience, patients’ anxiety or fear generally arises from concerns about potential platelet depletion following the COVID-19 infection, which could lead to organ bleeding or increased difficulty in home care.

Platelet ratios (e.g., PLR) are increasingly recognized as inexpensive and readily available biomarkers of inflammation and immune activity (42, 43). In our cohort study, PLR showed a trend of initially increasing, then decreasing, and finally returning to baseline levels. This is similar to the results reported in other ITP literature (44, 45). However, there was little change in NLR and MLR before and after COVID-19 infection, which requires more data for verification. In the context of ITP and COVID-19, these ratios may serve as early indicators of disease flare or remission, be associated with psychological stress levels, and provide a dual diagnostic tool. Additionally, they may improve risk stratification and facilitate personalized care plans. By emphasizing platelet ratios, this study may bridge the fields of clinical hematology and psychosomatic medicine, potentially influencing guidelines for the management of ITP patients during viral outbreaks.

Honestly, our study has some limitations, because of the difficulties of a retrospective design, the sample size is small; second, there may be selection bias and reliance on self-reported psychological data. We will conduct a prospective, multicenter study to examine the role of specific cytokines in transient platelet increases, as well as the interaction between hematological markers and psychiatric symptoms. Additionally, we will explore the long-term platelet behavior after viral infection in ITP patients.

In conclusion, some ITP patients show a transient increase in platelet count followed by a gradual decrease to baseline levels during the spread of the COVID-19. Moreover, due to the less understanding of platelet changes after COVID-19 infection, ITP patients show significant concerns in fare and anxiety. Although the period of widespread COVID-19 infection has passed, COVID-19 will continue to exist in the long term, which will continue to trouble ITP patients. Understanding the dynamic changes in platelet levels and psychological emotions of ITP patients before and after COVID-19 infection will help us better manage ITP patients physically and psychologically. Given the study’s limitations, the conclusions of this paper require further prospective research to be validated.

## Data Availability

The raw data supporting the conclusions of this article will be made available by the authors, without undue reservation.
