# Supplementary material for: Dynamic changes in platelet counts and psychological state in ITP patients after COVID-19 infection
Source: Front Med (Lausanne). 2025 Mar 14;12:1485418. doi: 10.3389/fmed.2025.1485418 (PMC11949926; doi:10.3389/fmed.2025.1485418)
Supplement: Supplementary file 1 [file Table_1.DOCX]

**Comparison of characteristics between healthy individuals and those with ITP**

| Variables | Total (n = 159) | Groups | | P |
| --- | --- | --- | --- | --- |
|  |  | ITP (n = 90) | Healthy (n = 69) |  |
| Fever<38℃, n (%) |  |  |  | 0.048 |
| no | 119 (74.8) | 62 (68.9) | 57 (82.6) |  |
| yes | 40 (25.2) | 28 (31.1) | 12 (17.4) |  |
| Fever(38.1～38.9)℃, n (%) |  |  |  | 0.146 |
| no | 91 (57.2) | 56 (62.2) | 35 (50.7) |  |
| yes | 68 (42.8) | 34 (37.8) | 34 (49.3) |  |
| Fever≥39℃, n (%) |  |  |  | 0.464 |
| no | 132 (83.0) | 73 (81.1) | 59 (85.5) |  |
| yes | 27 (17.0) | 17 (18.9) | 10 (14.5) |  |
| Cough, n (%) |  |  |  | 0.489 |
| no | 46 (28.9) | 28 (31.1) | 18 (26.1) |  |
| yes | 113 (71.1) | 62 (68.9) | 51 (73.9) |  |
| Throat pain, n (%) |  |  |  | 0.985 |
| no | 69 (43.4) | 39 (43.3) | 30 (43.5) |  |
| yes | 90 (56.6) | 51 (56.7) | 39 (56.5) |  |
| Chest tightness, n (%) |  |  |  | 0.256 |
| no | 137 (86.2) | 80 (88.9) | 57 (82.6) |  |
| yes | 22 (13.8) | 10 (11.1) | 12 (17.4) |  |
| Anorexia, n (%) |  |  |  | 0.274 |
| no | 82 (51.6) | 43 (47.8) | 39 (56.5) |  |
| yes | 77 (48.4) | 47 (52.2) | 30 (43.5) |  |
| Nausea and vomiting, n (%) |  |  |  | 0.19 |
| no | 121 (76.1) | 65 (72.2) | 56 (81.2) |  |
| yes | 38 (23.9) | 25 (27.8) | 13 (18.8) |  |
| Diarrhea, n (%) |  |  |  | 0.256 |
| no | 137 (86.2) | 80 (88.9) | 57 (82.6) |  |
| yes | 22 (13.8) | 10 (11.1) | 12 (17.4) |  |
| Nasal obstruction, n (%) |  |  |  | 0.157 |
| no | 77 (48.4) | 48 (53.3) | 29 (42) |  |
| yes | 82 (51.6) | 42 (46.7) | 40 (58) |  |
| Hypogeusia, n (%) |  |  |  | 0.829 |
| no | 96 (60.4) | 55 (61.1) | 41 (59.4) |  |
| yes | 63 (39.6) | 35 (38.9) | 28 (40.6) |  |
| Myodynia, n (%) |  |  |  | 0.65 |
| no | 82 (51.6) | 45 (50) | 37 (53.6) |  |
| yes | 77 (48.4) | 45 (50) | 32 (46.4) |  |
| Dizziness, n (%) |  |  |  | 0.439 |
| no | 82 (51.6) | 44 (48.9) | 38 (55.1) |  |
| yes | 77 (48.4) | 46 (51.1) | 31 (44.9) |  |
| Hypomnesis, n (%) |  |  |  | 0.174 |
| no | 140 (88.1) | 82 (91.1) | 58 (84.1) |  |
| yes | 19 (11.9) | 8 (8.9) | 11 (15.9) |  |
| Dysacusis, n (%) |  |  |  | 0.317 |
| no | 155 (97.5) | 89 (98.9) | 66 (95.7) |  |
| yes | 4 ( 2.5) | 1 (1.1) | 3 (4.3) |  |
| Conjunctivitis, n (%) |  |  |  | 0.634 |
| no | 155 (97.5) | 87 (96.7) | 68 (98.6) |  |
| yes | 4 ( 2.5) | 3 (3.3) | 1 (1.4) |  |
| Other.symptoms, n (%) |  |  |  | 0.317 |
| no | 155 (97.5) | 89 (98.9) | 66 (95.7) |  |
| yes | 4 ( 2.5) | 1 (1.1) | 3 (4.3) |  |
| Days of fever, Median | 2.0 (1.0, 3.0) | 2.0 (1.0, 3.0) | 2.0 (2.0, 3.0) | 0.479 |
| Viral RNA turned to be negative, n (%) |  |  |  | 0.746 |
| (≤7days) | 44 (27.7) | 24 (26.7) | 20 (29) |  |
| (>7days) | 115 (72.3) | 66 (73.3) | 49 (71) |  |
| The degree of pain, Median (IQR) | 3.0 (2.0, 4.0) | 2.0 (1.0, 3.0) | 4.0 (3.0, 7.0) | < 0.001 |
| BMI, Mean ± SD | 27.1 ± 4.6 | 30.2 ± 2.5 | 22.9 ± 3.4 | < 0.001 |
