# Supplementary material for: Dynamic changes in platelet counts and psychological state in ITP patients after COVID-19 infection
Source: Front Med (Lausanne). 2025 Mar 14;12:1485418. doi: 10.3389/fmed.2025.1485418 (PMC11949926; doi:10.3389/fmed.2025.1485418)
Supplement: Supplementary file 2 [file Table_2.DOCX]

**Supplementary file 2**

**Table 1 Multivariate analysis of the effects of different variables on platelets**

Y=P0(Platelets in ITP patients before infection with COVID-19)

| Variable | n | crude. Coefficient (95%CI) | crude.P value | adj.Coefficient (95%CI) | adj.P value |
| --- | --- | --- | --- | --- | --- |
| Age | 90 | -0.78 (-1.71~0.14) | 0.1 | 0.42 (-1.1~1.94) | 0.592 |
| Gender |  |  |  |  |  |
| male | 15 | 0(Ref) |  | 0(Ref) |  |
| Female | 75 | 11.2 (-27.54~49.94) | 0.572 | 25.45 (-17.03~67.92) | 0.244 |
| BMI | 90 | -2.47 (-6.06~1.12) | 0.181 | -1.91 (-5.83~2.02) | 0.344 |
| <18 | 8 | 0(Ref) |  | 0(Ref) |  |
| 18-24 | 54 | -27.85 (-78.96~23.26) | 0.288 | -13.5 (-78.27~51.27) | 0.684 |
| >24 | 28 | -50.84 (-104.92~3.24) | 0.069 | -34.16 (-127.33~59.01) | 0.475 |
| Treatment of ITP |  |  |  |  |  |
| no | 63 | 0(Ref) |  | 0(Ref) |  |
| yes | 27 | -24.56 (-55.7~6.58) | 0.126 | -26.47 (-61.74~8.79) | 0.145 |
| Clearance. Time | 90 | -7.78 (-13.33~-2.23) | 0.007 | -7.39 (-13.53~-1.26) | 0.021 |
| Vaccination |  |  |  |  |  |
| no | 51 | 0(Ref) |  | 0(Ref) |  |
| yes | 39 | -6.62 (-35.78~22.53) | 0.657 | -10 (-41.82~21.81) | 0.54 |

**Table 2 Multivariate analysis of the effects of different variables on platelets**

Y=P1(Platelets in ITP patients 1 week after infection with COVID-19)

| Variable | n | crude. Coefficient (95%CI) | crude.P value | adj.Coefficient (95%CI) | adj.P value |
| --- | --- | --- | --- | --- | --- |
| Age | 90 | 0.45 (-1.83~2.73) | 0.701 | 2.48 (-1.12~6.08) | 0.181 |
| Gender |  |  |  |  |  |
| male | 15 | 0(Ref) |  | 0(Ref) |  |
| Female | 75 | 57.69 (-35.98~151.37) | 0.231 | 48.25 (-52.26~148.76) | 0.35 |
| BMI | 90 | 3.69 (-5.11~12.49) | 0.414 | 0.28 (-9.01~9.57) | 0.953 |
| <18 | 8 | 0(Ref) |  | 0(Ref) |  |
| 18-24 | 54 | 77.57 (-48.12~203.27) | 0.23 | 70.8 (-76.75~218.34) | 0.35 |
| >24 | 28 | 35.85 (-97.16~168.86) | 0.599 | -49.09 (-261.33~163.14) | 0.652 |
| Treatment of ITP |  |  |  |  |  |
| no | 63 | 0(Ref) |  | 0(Ref) |  |
| yes | 27 | -60.39 (-136.16~15.38) | 0.122 | -52.46 (-136.28~31.37) | 0.224 |
| Clearance. Time | 90 | 5.67 (-8.36~19.69) | 0.431 | 5.16 (-9.86~20.18) | 0.503 |
| Vaccination |  |  |  |  |  |
| no | 51 | 0(Ref) |  | 0(Ref) |  |
| yes | 39 | 4.72 (-66.31~75.75) | 0.897 | -6.78 (-82.25~68.69) | 0.861 |
